# PERCEIVED STRESS, SOCIAL SUPPORT, AND DRY MOUTH AMONG U.S. OLDER CHINESE ADULTS

**DOI:** 10.1093/geroni/igz038.119

**Published:** 2019-11-08

**Authors:** Weiyu Mao, Weiyu Mao, Yiwei Chen, Bei Wu, Shaoqing Ge, Wei Yang, Iris Chi, XinQi Dong

**Affiliations:** 1 University of Nevada, Reno, Reno, Nevada, United States; 2 Bowling Green State University, Bowling Green, Ohio, United States; 3 New York University, New York, New York, United States; 4 Duke University, Durham, North Carolina, United States; 5 University of Southern California, Los Angeles, California, United States; 6 Rutgers, The State University of New Jersey, New Brunswick, New Jersey, United States

## Abstract

Dry mouth is a common condition among older adults that negatively influences oral health, general health, and quality of life. The role of psychosocial factors in oral health conditions and diseases remains largely unknown. We examined the relationship between perceived stress and dry mouth among US older Chinese adults and further investigated the moderating role of social support from different sources in the relationship. Data came from baseline of the Population Study of Chinese Elderly in Chicago between 2011 and 2013 (N = 3,157). Stepwise logistic regression models with interaction terms were used. More perceived stress was significantly associated with a higher likelihood of reporting dry mouth. Friend support was protective against dry mouth. The effect of perceived stress on dry mouth varied by levels of family and friend support. To prevent or reduce dry mouth, interventions need to consider perceived stress and social support in this growing population.

